# Umbilical cord blood‐derived cytotoxic T lymphocytes target melanoma via HLA‐A2‐restricted tumour antigens

**DOI:** 10.1002/ctm2.70444

**Published:** 2025-10-15

**Authors:** Jiaji Liang, Xiao Jiang, Xi He, Zhiqin Dong, Jinqiang Lu, Shuxian Jiang, Hengyu Du, Haoran Mao, Songtao Luo, Xifeng An, Hongwei Liu

**Affiliations:** ^1^ Department of Plastic Surgery of the First Affiliated Hospital of Jinan University Institute of New Technology of Plastic Surgery of Jinan University Key Laboratory of Regenerative Medicine of Ministry of Education Guangzhou China; ^2^ Department of Aesthetic Medicine Liwan Central Hospital Guangzhou China

**Keywords:** antigen‐specific TCR, cancer immunotherapy, gp100, MART1, melanoma, umbilical cord blood

## Abstract

**Background:**

Melanoma is recognized as a highly malignant cancer with a generally poor prognosis, underscoring the critical need for effective therapeutic strategies. Adoptive cell therapy has emerged as a promising modality to improve treatment outcomes in melanoma. For endogenous cell therapy (ECT), peripheral blood (PB) has traditionally served as the primary cell source. However, the potential of umbilical cord blood (UCB) as an alternative source for ECT remains unclear. Furthermore, the repertoire of TCRs remains limited. These deficiencies impede the optimization and broader application of ECT for melanoma, highlighting the necessity for focused investigations to resolve these issues.

**Methods:**

To evaluate the effects of HLA‐A2 restricted antigen‐specific CD8⁺ T cells on melanoma cells, the cytotoxic activity of CD8⁺ T cells derived from UCB and PB were conducted in vivo and in vitro assays. Single‐cell RNA sequencing combined with TCR V(D)J sequencing was employed to characterize cellular composition and quantify the frequencies of specific TCR clonotypes. The generation probability and peripheral occurrence probability of antigen‐specific CD8⁺ TCR sequences from UCB and PB were computed using the Simple Olga Sonia algorithm. Finally, molecular docking simulations were conducted to predict the binding affinity between isolated TCRs and pMHC.

**Results:**

No significant differences were observed in the cytotoxic effects mediated by antigen‐specific CD8⁺ T cells derived from UCB versus PB. Phenotypic analysis revealed that PB‐derived antigen‐specific CD8⁺ T cells were predominantly effector and proliferating cells, whereas those from UCB consisted largely of memory cells. TCR sequencing identified a greater diversity of antigen‐specific TCR clonotypes in PB, meanwhile UCB‐derived TCRs exhibited strong pMHC binding. Molecular docking simulations confirmed high binding affinity between pMHC and TCR clones isolated from both sources.

**Conclusions:**

Antigen‐specific CD8⁺ T cells from UCB and PB display comparable cytotoxic efficacy against melanoma, albeit with distinct compositional profiles of antigen‐specific CD8⁺ T cell subsets. Candidate TCRs can be effectively activated by the tumor‐associated antigens MART1 and gp100. This activation promotes the expansion of the available TCR repertoires, thereby mitigating the previous constraint of a limited TCR library.

**Key points:**

- Endogenous T‐celltherapy for melanoma has used adult peripheral blood as the cell source andachieved certain results, but the cellular components of effector T cells werenot clear.
- At the same time, it was not clear whether umbilical cord bloodcould be used as a cell resource bank for endogenous T‐cell therapy.
- This studydemonstrated that T cells from umbilical cord blood can be used as a cellresource bank for endogenous T‐cell therapy.
- It further clarified that thereare different components of antigen‐specific T cells in umbilical cord bloodand peripheral blood.
- Antigen‐specific T cells from peripheral blood are mainlyproliferative T cells, while those from umbilical cord blood are mainlyeffector T cells and memory T cells.
- Finally, TCR sequencing was used to obtainTCRs that can target melanoma, expanding the corresponding TCR database.

## Graphical abstract

Cord blood can be used as a cell bank for endogenous T‐cell therapy to treat melanoma patients. Compared with antigen‐specific T cells derived from peripheral blood, the main cell subsets of antigen‐specific T cells derived from umbilical cord blood are divided into memory effector cells, which may have a more durable anti‐tumour effect. In this study, TCR targeting melanoma was obtained by TCR sequencing, and the corresponding TCR database was expanded.

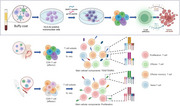

## 1 INTRODUCTION

Melanoma is a malignant tumour originating from melanocytes, characterised by aggressive, early lymphatic and haematogenous metastasis, and a poor prognosis. It most commonly occurs in Caucasian populations. Notably, although the incidence of many tumours has declined, the prevalence of melanoma has steadily increased, rising at a rate of 3%–7% over the past 50 years.^1^, ^2^, ^3^ Before the advent of targeted therapy and immunotherapy, surgical resection and radiotherapy were the primary therapeutic approaches, albeit with low efficacy.^4^, ^5^ Although targeted therapy and immunotherapy have demonstrated efficacy against melanoma, disparities between Chinese and Western melanoma populations, beginning with pathological types and encompassing molecular and immunological differences, influence the effectiveness of targeted therapies and immune checkpoint inhibitors in treating Chinese patients with melanoma.^6^, ^7^, ^8^, ^9^

Recently, new immunotherapy strategies and therapeutic targets have garnered interest in melanoma research. Melanoma is characterised by the high expression of cancer‐testis antigens (CTAs) and tumour‐associated antigens, which serve as immunotherapy targets.^10^, ^11^ MART1 and gp100 are typical melanoma antigens processed intracellularly with human leukocyte antigen (HLA) on the cell surface, and are also utilised as important melanoma therapeutic targets.^12^ T cells are crucial to anti‐tumour immunity due to their ability to directly kill tumour cells; consequently, transplantation of tumour antigen‐specific T cells is an effective cancer treatment strategy because of its sustained anti‐cancer response. This study reports that antigen‐specific CD8^+^ T cells derived from Umbilical cord blood mononuclear cells(UCBMCs) targeting MART1 and gp100 antigens successfully promoted an anti‐cancer response.^13^, ^14^, ^15^

For all patients, identifying a suitable T‐cell donor presents a significant challenge. UCBMCs offer an ideal alternative source of effector cells to HLA‐matched donor allogeneic cells, due to their unique properties: (i) they are abundant and noninvasive to collect from donors; (ii) they exhibit immune immaturity and high plasticity; (iii) they can survive long‐term storage while maintaining cell viability; (iv) they facilitate timely cell transplantation, shorten treatment cycles, and comply with ethical standards.^16^, ^17^ In cases of antigenic mismatch, the use of immune cells derived from UCBMC reduces the risk of graft‐versus‐host disease.^18^ These unique properties make UCBMC an ideal cell source for melanoma therapy. However, the composition of their cell subsets and the anti‐melanoma effects of CD8^+^ T cells derived from UCBMCs remain unclear.

## 2 MATERIALS AND METHODS

### 2.1 Clinical samples

Umbilical cord blood and peripheral blood samples, each containing 50 mL, were collected from healthy donors at the First Affiliated Hospital of Jinan University. Blood mononuclear cells were isolated using density gradient centrifugation with lymphocyte separation medium (GE). PBMC blasts were obtained from healthy female donors aged 26–32 years. UCBMC blasts were obtained from umbilical cords of women aged 29–35 years during labour. All donors were informed about the study and provided consent before blood collection.

### 2.2 Tumour cell lines

UCBMCs and PBMCs were harvested by leukapheresis after informed consent was obtained. HLA‐A2‐positive samples were detected by flow cytometry and stained with PE‐conjugated anti‐human HLA‐A2 antibody (Biolegend) to identify the HLA‐A2^+^ donors. Human melanoma cell lines A375 (HLA‐A2^+^, MART1^−^/gp100^−^), SK‐Mel‐5 (HLA‐A2^+^, MART1^+^/gp100^+^) were obtained from American Type Culture Collection (ATCC). T2 (ATCC) is an HLA‐A2^+^ human TAP‐deficient hybridoma cell line used as a target in a functional avidity assay.

### 2.3 Culture of dendritic cells

PBMCs and UCBMCs were suspended at a concentration of 2 × 10⁶ cells/mL in RPMI 1640 medium (Solarbio), supplemented with 100 U/mL penicillin and streptomycin (Solarbio), and 1% autologous serum. Subsequently, the cells were placed in sterile six‐well plates at 2 mL per well to separate plastic‐adherent (myeloid progenitor cells) and non‐adherent (lymphoid cells) populations. The separation was achieved by culturing for 1 h at 37°C. Myeloid progenitor cells were treated with GM‐CSF (1000 U/mL) (Xiamen Amoytop Biotech, CN) and IL‐4 (500 U/mL) (R&D Systems) for 1 week to promote immature dendritic cell (iDC) differentiation. The iDCs were analysed by flow cytometry for markers CD80, CD86 and HLA‐DR. Subsequently, the iDCs were matured for 1 day using a cytokine cocktail containing TNF‐α (50 ng/mL) (Shanghai Weike Biopharmaceutical, CN). CD80, CD86 and HLA‐DR markers were also assessed by flow cytometry in the mature dendritic cells (mDCs). Typically, mDCs were used fresh for initial stimulation; however, any excess DCs were frozen and frequently utilised in subsequent stimulations.^19^

### 2.4 Stimulation of MART1 and gp100‐specific T cells

Autologous monocyte‐derived DCs (2 × 10^6^) were pulsed for 4 h with 40 µg/mL of A2‐restricted synthesised peptide epitopes, MART1_27–35_ (AAGIGILTV) and gp100_154–162_ (KTWGQYWQV) (GenScript), in KBM 581 medium (Corning) supplemented with 5% autologous serum. These mDCs were then co‐cultured with autologous T cells at a 10:1 ratio (T cells: mDCs) in 48‐well plates. Each plate contained 2 × 10^7^ T cells and 2 × 10^6^ DCs, distributed equally among the 48 wells. Interleukin 21 (IL‐21) at 30 ng/mL, interleukin 2 (IL‐2) at 12.5 U/mL, and interleukin 7 (IL‐7) at 10 ng/mL were added on Days 1, 3 and 5, respectively.^18^ Two stimulations with peptide‐pulsed mDCs were conducted 7 days apart. IL‐2 (50 IU/mL) and IL‐7 (10 ng/mL) were added 1 day after the second stimulation to facilitate further expansion of activated antigen‐specific CD8^+^ T cells. Stimulations for gp100 and MART1 antigen‐specific CD8^+^ T cells followed the same procedure, using the appropriate peptides gp100_154–162_ and MART1_27–35_, respectively. After two cycles of stimulation at weekly intervals, CD8^+^ T cells were cloned and expanded for in vitro testing.^20^, ^21^, ^22^

### 2.5 Sorting and expansion

PE‐labelled MART‐1 MHC tetramers and gp100 MHC tetramers were produced according to established protocols. Streptavidin conjugated with a PE‐fluorophore (Biolegend) was mixed with peptide‐exchanged monomers (GenScript, CN) at a final molar ratio of 30:3.3 (Monomer:Streptavidin) and incubated in the dark on ice for 30 min. Subsequently, 2.4 µL of blocking solution, containing 1.6 µL 50 mM biotin (Thermo Fisher) and 198.4 µL PBS (Gibco), was added and incubated overnight at 4°C–8°C. Following two stimulations, aliquots from each well were stained with HLA‐A2 MHC tetramer complexed with the gp100_154–162_ and MART1_27–35_ peptides. For analysis, 1 × 10^5^ cells in PBS containing 2% FBS (Gibco) were first stained with the peptide tetramer‐PE for 1 h at room temperature and then stained with APC‐anti‐CD8 (BD Pharmingen) for 20 min at 4°C. After washing with PBS, the cells were resuspended in PBS with 2% FBS. Flow cytometry was conducted using a FACS Aria III (BD Pharmingen) to analyse antigen‐specific CD8^+^ T cells. Wells containing more than 0.5% tetramer‐positive cells were pooled and sorted with a BD FACS Aria SORP cell sorter (BD Pharmingen). The sorted tetramer‐positive T cells were plated in 96‐well round‐bottom plates coated with an anti‐CD3 antibody (OKT3, Ortho Biotech) at 30 ng/mL, and incubated in PBS at 4°C overnight. These antigen‐specific T cells were expanded in 14‐day cycles by adding feeder cells and rhIL‐2 at 500 U/mL every 2–3 days. After expansion, the T cells were restained with APC‐anti‐CD8 and PE‐gp100_154–162_/MART1_27–35_ tetramers to assess the enrichment of tetramer‐positive cells.^23^, ^24^ Finally, cell count analysis was conducted on the antigen‐specific CD8^+^ T cells to determine the cell expansion efficiency. All measurements were performed in triplicate and repeated for data confirmation.

### 2.6 In vitro peptide dose titration assay of antigen‐specific CD8^+^ T cells against T2 cells

For the peptide dose titration assay, T2 cells (1 × 10^5^ cells/well) were pulsed with a peptide at concentrations ranging from 10^1^ to 10^7^ pg/mL for 1 h and subsequently washed with PBS. Antigen‐specific CD8^+^ T cells (1 × 10^5^ cells/well, effector cells) were incubated with APC‐anti‐CD8 for 20 min at 4°C. T2 cells were then co‐cultured with the effector cells for 3 h at 37°C with 5% CO_2_. FITC‐DEVD‐FMK (Abcam) was added to the RPMI 1640 medium for another hour to bind to activated caspase‐3 in apoptotic cells. The expression of FITC in T2 cells was measured by fluorescence detection. All measurements were performed in triplicate and repeated for data confirmation.^25^

### 2.7 In vitro cytotoxicity assay of antigen‐specific CD8^+^ T cells against human melanoma cells

Target cells from A375 and SK‐Mel‐5 melanoma cell lines, at a density of 2 × 10^5^ cells per well, were co‐cultured with effector cells for 3 h at 37°C and 5% CO_2_. FITC‐DEVD‐FMK was added for an additional hour in EMEM medium (MEM supplemented with NEAA, 10% FBS and 1% PS). Antigen‐specific CD8^+^ T cells were introduced at varying effector‐to‐target (E:T) ratios: 10:1, 5:1, 2.5:1, 1.25:1 and 1:1.6. Subsequently, the expression of FITC in T2 cells was assessed using fluorescence detection. All measurements were conducted in triplicate and repeated to validate the data.^26^

### 2.8 IFN‐γ cytokine detection

A375 and Sk‐Mel‐5 cells were seeded in a 96‐well round‐bottom plate in triplicate at a density of 10⁴ cells per well. Antigen‐specific CD8^+^ T cells were co‐cultured with the tumour cells at a density of 10⁵ cells per well, incubating them overnight. On the following day, 10 µL of supernatants were collected and analysed using a human IFN‐γ ELISA kit (JINGMEI Biotech, CN), following the manufacturer's protocol. The absorbance was measured at 450 nm using a spectrophotometer (BioTek Instruments). All measurements were conducted in triplicate and repeated to validate the data.^27^

### 2.9 In vivo mouse model

In this study, A375 and SK‐Mel‐5 cells were transduced with the pLenti‐EF1a‐EGFP‐CMV‐Luc2 lentivirus (OBiO Technology), and positively transduced cells were selected using 5 µg/mL puromycin over 5 days. There were 64 male BALB/c nude mice at the age of 5 weeks of sexual maturity, weighing 18–22 g, purchased from the Guangdong Medical Laboratory Animal Center (Certificate Number SCXK [Yue] 2023‐0204). The study was approved and overseen by Jinan University Application for Laboratory Animal Ethical Review (20230309‐0004). After numbering, the experimental animals were divided into 16 groups using random numbers. The nude mice were housed in a vivarium within individually ventilated cages, with access to food and sterilised water. For subcutaneous injection, each nude mouse was injected with 5 × 10^6^ A375 cells in a 100 µL volume of PBS/Matrigel (1:1; Matrigel: Yeasen Biotech). For the treatment of cell‐derived xenograft tumours, mice with similarly sized tumours were randomly divided into groups of three. In vivo imaging, nude mice were anaesthetised with 0.3% sodium pentobarbital and 10 µL per gram of bodyweight was injected intraperitoneally. Each mouse received intravenous transplants of either 1 × 10^7^ tumour antigen‐specific CD8^+^ T cells (MART‐1 and gp100 derived from PBMCs and UCBMCs) or control T cells (non‐specific T cells derived from PBMCs and UCBMCs), administered twice to the corresponding groups. IL‐2 (2 × 10^5^ IU per mouse) was intraperitoneally injected concurrently. Fluorescence intensity was measured weekly using live imaging techniques and calculated with the formula: Radiance = *p*/s/cm^2^/sr. When the size of the subcutaneous mass exceeded 2.5 × 2.5 cm, all animals were euthanased.^20^

### 2.10 The cell kinetics experiment

For the animal models in which melanoma cells A375 and Sk‐Mel‐5 successfully formed tumours under subcutaneous implantation, antigen‐specific CD8^+^ T cells from PBMCs or UCBMCs were intravenously injected. The animals were euthanased on the third and seventh days, and the tumour tissues were surgically removed and dissociated. The infiltrating lymphocytes within the tumour tissues were extracted for HLA‐A2 MHC tetramer complexed with the gp100_154–162_ and MART1_27–35_ peptides staining. All measurements were conducted in triplicate and repeated to validate the data.^28^

### 2.11 Single‐cell RNA sequencing

Antigen‐specific CD8^+^ T cells were sorted using the FACS Aria III flow cytometer with PE‐tetramers and APC‐anti‐CD8 antibodies. Single cell suspensions were counted using both the Cellometer K2 fluorescent viability cell counter (Nexcelom) and a haemocytometer, and were then adjusted to a concentration of 1000 cells/µL. Subsequently, droplet‐encapsulation scRNA‐seq experiments were conducted, each involving 10,000 viable single cells loaded onto the Chromium Controller (10X Genomics). Following droplet encapsulation, cDNA synthesis and amplification, sequencing libraries were generated utilising the Chromium Single Cell 5′ Feature Barcode Library Kit, the Chromium Single Cell 5′ Library & Gel Bead Kit, and the Chromium Single Cell V(D)J Enrichment Kit (Human T Cell) from 10X Genomics, in accordance with the manufacturer's instructions. Libraries from each channel (up to eight channels) were multiplexed and sequenced on an Illumina NovaSeq 6000.^29^

### 2.12 ScRNA‐seq data processing

Raw base call (BCL) files were analysed using CellRanger version 2.1.1. The ‘mkfastq’ command generated FASTQ files, while the ‘count’ command produced raw gene‐barcode matrices aligned with the 10X Genomics GRCh38 Ensembl build 84 genome, version 1.2.0. Data from all samples were combined in R version 3.5.2 using the Read10X function from the Seurat package version 2.3.3, resulting in an aggregated Seurat object. Cells with unique molecular identifiers (UMIs) greater than 500 and mitochondrial content less than 10% were retained during filtering. Data were normalised using the ‘LogNormalize’ method with a scale factor of 10,000. Variably expressed genes were identified with normalised expression between 0.125 and 3, and a quantile‐normalised variance exceeding 0.5. Clustering was conducted using 40 PCA components and a resolution parameter set to 10. The original Louvain algorithm was utilised for modularity optimisation. Related genes were annotated with data generated from the FindAllMarkers() Seurat differential expression.^29^

### 2.13 SCENIC and cell–cell communication analysis

The pySCENIC (0.9.9 + 2.gcaded79) algorithm was run on a normalised expression matrix of the corresponding samples. The GRNboost2 (arboreto 0.1.5) method was utilised for gene regulatory network reconstruction. The cisTarget Human motif database v9 (https://resources.aertslab.org/cistarget/motif2tf/motifs‐v9‐nr.hgnc‐m0.001‐o0.0.tbl) was used for enrichment of gene signatures and pruned for targets from this signature based on cis‐regulatory cues with default settings. The ‘aucell’ positional argument was utilised to find enrichment of regulons across single cells. The resulting matrix was *z*‐scored using the standardise () function from the psycho (0.4.9) R package, and the results were visualised using a heatmap with hierarchical clustering. We used the CellChat R package to input expression data and cell type information. This allowed us to identify potential communication signals between different cell types using the built‐in ligand–receptor interaction database. Each cell type was evaluated as a source and target of signals to other cell types. We constructed a cell communication network and calculated the role of each cell type within it.^29^

### 2.14 Single‐cell V(D)J sequencing analysis

The raw BCL files for the T cells library were analysed using CellRanger (version 2.2.0). The ‘mkfastq’ command was employed to generate FASTQ files, while the ‘vdj’ command was used to produce sequence annotations. These files were aligned to the GRCh38 Ensembl build genomes and included a 10X‐specific gene addendum and a blacklist of transcript IDs (version 2.0.0). The raw data from each sample, derived from the ‘all_contig_annotations.csv’ output, were intersected with T and B cells that had been previously filtered using Seurat. Additional filtering was applied by including only clonotypes labelled as having ‘productive’, ‘high_confidence’ and ‘is_cell’ attributes set to true. Clonotypes were subsequently grouped by ‘raw_clonotype_id’ for determining their abundance.^29^

### 2.15 Probability estimation and generation of T‐cell receptors

The Igor, OLGA and Sonia algorithms enable inference of V(D)J recombination generation and selection in vivo from TCR sequencing data. SOS (https://sites.google.com/view/statbiophysens/sos) is built upon the IGOR, OLGA and SONIA software frameworks to evaluate the generation probability (Pgen) and post‐selection probability (Ppost) of species‐specific human T‐cell receptor sequences. Pgen depends on the sequence's productivity and models V(D)J recombination in the absence of selection pressures. Ppost incorporates these selection pressures and thus better reflects the sequence's frequency within the peripheral repertoire. Enter the CDR3 sequence (amino acid) as well as the V and J segments in the network interface. We statistically analysed the Pgen and Ppost values for the top 15 productive V(D)J recombinations identified by sequencing. Furthermore, we characterised the distribution of the selection factor Q (Q = Ppost/Pgen). A higher Q value indicates a sequence that is more likely to have undergone positive selection within the periphery.^30^

### 2.16 Prediction of antigen‐specific TCRs

The TCRmodel2 code, adapted from AlphaFold v2.3.0 (https://github.com/piercelab/tcrmodel2), demonstrates comparable or superior accuracy to AlphaFold and other methods in modelling TCR–peptide–MHC complexes based on benchmarking tests. The input files for TCRmodel2 include TCR‐seq‐derived TCR CDR3β data matched through cell barcodes. We utilised TCRmodel2 to assess the credibility of the largest number of cloned T cells using the MART1 and gp100 antigen‐specific CD8^+^ T‐cell TCR‐seq data mentioned above. We recommend using the web server for generating predictions (https://tcrmodel.ibbr.umd.edu/).^31^, ^32^

### 2.17 Statistical analysis

All statistics were performed by GraphPad Prism 9, SPSS 26.0 software and the R statistical package. All in vitro experiments consisted of three or more biological replicates per experimental group and were represented as individual data points. In vivo studies consisted of three or four mice per group. Statistical tests were performed on data from independent biological replicates. For comparisons between two groups, a two‐way ANOVA test was applied. For comparing three groups, one‐way ANOVA with Bonferroni post hoc test was applied. One‐way ANOVA with Dunnett post hoc test was used for comparing a control group with two experimental groups. Seurat differential expression analysis was done using the default two‐sided non‐parametric Wilcoxon rank‐sum test with Bonferroni correction. *p*‐value less than 0.05 was considered to be significant; *p <0.05; ***p <0.001.

## 3 RESULTS

### 3.1 Isolation of UCBMC and PBMC for HLA‐A2 cell identification and dendritic cell phenotyping

UCBMCs and PBMCs in the buffy coat primarily consist of monocytes and lymphocytes (Figure S1A). Mononuclear cells from the buffy coat were collected for HLA‐A2 flow cytometry analysis. Compared to the control group without negative staining, the fluorescence intensity of HLA‐A2 in the experimental group ranged between 10^4^ and 10^5^, indicating strong positivity and confirming that the screened cell samples were HLA‐A2‐positive (Figure S1B). This result provides a phenotype basis for subsequent experiments.

### 3.2 Induction of monocyte‐derived dendritic cells

The morphology of adherent monocytes in UCBMCs and PBMCs altered 7 days post‐induction with IL‐4 and GM‐CSF, differentiating into iDCs. MDCs were obtained after a day stimulation with TNF‐α (Figure S1E). Flow cytometry using antibodies against CD80, CD86 and HLA‐DR surface markers determined dendritic cell phenotype post‐induction. Results indicated a significant increase in CD80‐ and CD86‐positive cells in immature dendritic cells compared to the unstained negative control (green). Moreover, CD80 and CD86 expression in mature dendritic cells also increased (blue) (Figure S1C). The expression pattern of HLA‐DR in mature dendritic cells mirrored that of CD80 and CD86 (Figure S1D).

### 3.3 Activation, proliferation and detection of antigen‐specific CD8^+^ T cells

**FIGURE 1:**
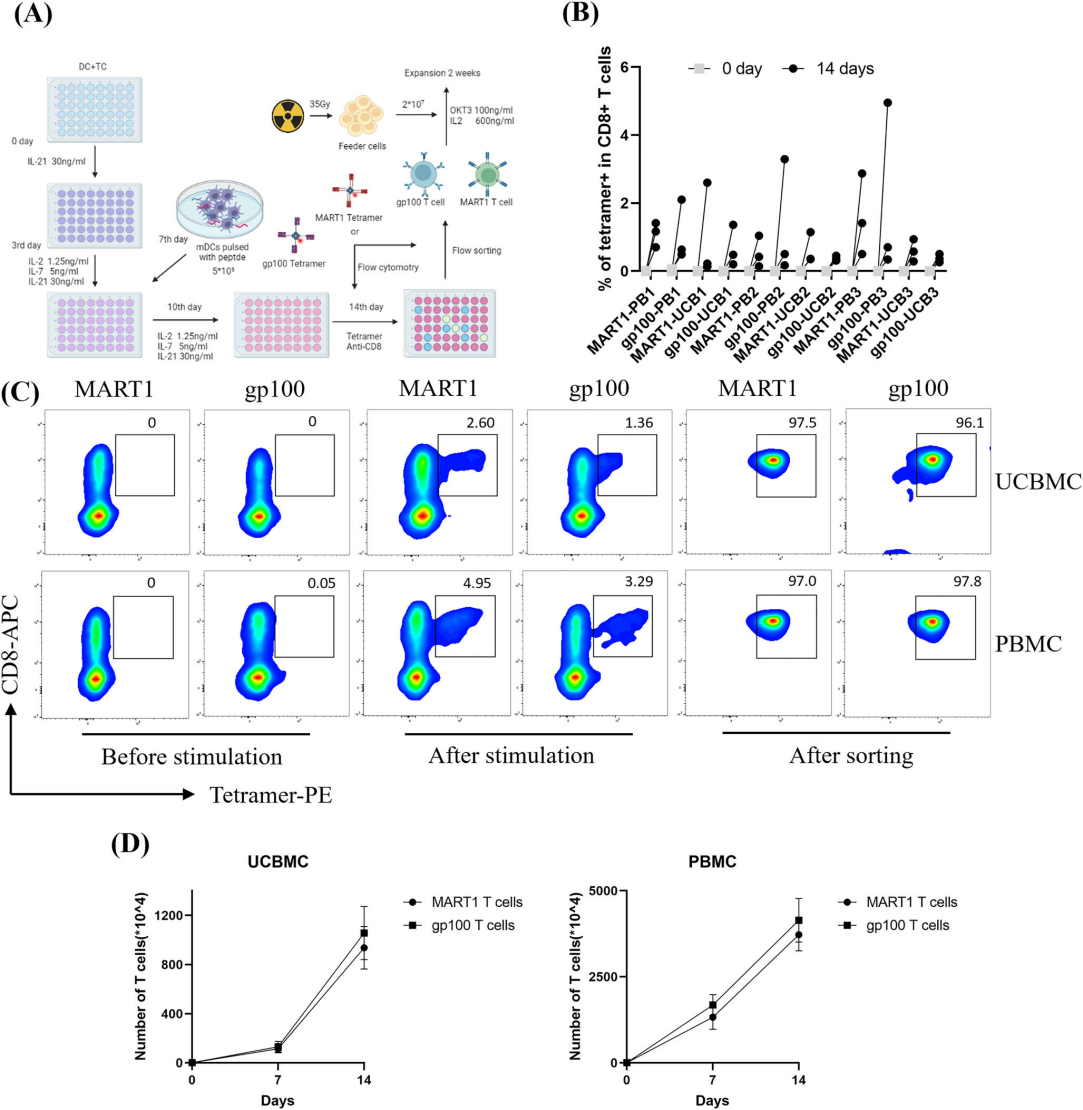
Culture of MART1 and gp100 antigen‐specific CD8^+^ T cells from UCBMCs and PBMCs. (A) This diagram illustrates the induction and expansion process of antigen‐specific T cells. (B) It presents the changes in proportions of MART1 and gp100 antigen‐specific T cells, which are induced by HLA‐A2 from PBMCs and UCBMCs, after co‐culture of 14 days. Here, the grey circle indicates the tetramer‐positive condition on Day 0, while the black circle indicates the tetramer‐positive condition on Day 14. (C) Before peptide stimulation, the flow cytometry analysis showed that the proportion of MART1 and gp100 tetramer and CD8 double‐positive cells was between 0% and 0.05%. After 14 days of co‐culture, flow cytometry with APC‐CD8 and PE‐MART1‐tetramer/PE‐gp100‐tetramer double staining revealed that the proportion of antigen‐specific CD8^+^ T cells from cord blood fluctuated between 0% and 3%, while those from peripheral blood ranged from 0% to 5%. Following separation and amplification, tetramer and CD8 double staining was performed on selected cells. The double‐positive ratio of antigen‐specific CD8^+^ T cells from both sources exceeded 95% after sorting. *N* = 3. (D) Cell growth was quantified over time, with absolute cell counts determined on Day 7 and Day 14. On Day 14, UCBMC‐derived CD8⁺ T cells specific for MART1 and gp100 antigens demonstrated significant expansion, reaching an average cell count of 1 × 10^7^ to 1.5 × 10^7^, with a corresponding fold expansion ranging from 600 to 850. Similarly, CD8⁺ T cells specific for MART1 and gp100 antigens derived from PBMCs exhibited a fold expansion of 450–580 when cultured in the presence of feeder cells at a density of 3 × 10^7^ to 5 × 10^7^. Data are represented as the mean ± standard deviation from three independent experiments.

A total of 1.2 × 10^7^ mixed cells derived from UCBMCs and autologous T cells were cultured in 48‐well plates. Antigen‐specific CD8^+^ T cells capable of binding to either the MART1‐MHC and gp100‐MHC complexes were activated and exponentially expanded post co‐culture (Figure S1F). Tetramer/CD8^+^ detection was conducted on cultured T cells induced by UCBMCs and PBMCs, resulting in detection of tetramer/CD8^+^ double‐positive T cells in at least two to five wells of the induced mixed cell cultures, with MART1 and gp100 antigen‐specific CD8^+^ T‐cell frequencies ranging from 0.13% to 2.79% from UCBMC and from 0.11% to 5.03% from PBMCs (Figure 1B).

### 3.4 Isolation and amplification of MART1 and gp100 antigen‐specific CD8^+^ T cells

The average frequency of antigen‐specific CD8^+^ T cells was 0.29% from UCBMCs and 1.49% from PBMCs. Post‐sorting data reveal that approximately 18,000–25,000 antigen‐specific CD8^+^ T cells could be selected from UCBMCs, while 70,000–85,000 could be selected from those derived from PBMCs. The screened cells underwent tetramer/CD8^+^ staining again, and the proportion of antigen‐specific CD8^+^ T cells of MART1 and gp100 from UCBMCs and PBMCs was over 95% (Figure 1C). T‐cell proliferation exhibited a density‐dependent pattern, with significant expansion observed after 7 days of stimulation using OKT3 and high‐dose IL‐2 following the addition of a feeder layer (cell density: 2 × 10^6^ to 1 × 10^7^ cells/well in 12‐well plates). By Day 14, the average number of MART1‐ and gp100‐specific CD8⁺ T cells expanded from UCBMCs increased from 1 × 10^7^ to 1.5 × 10^7^ cells, representing a 600–850‐fold amplification. Similarly, MART1‐ and gp100‐specific CD8⁺ T cells derived from PBMCs using a feeder layer of 3 × 10^7^ to 5 × 10^7^ cells showed a 450–580‐fold amplification (Figure 1D). These T cells were in circular suspension and exhibited uniform size (8–15 µm) (Figure S1G,H).

### 3.5 Antigen‐specific CD8^+^ T cells derived from UCBMC and PBMC have cytotoxic effects on melanoma cells and T2 cells

**FIGURE 2:**
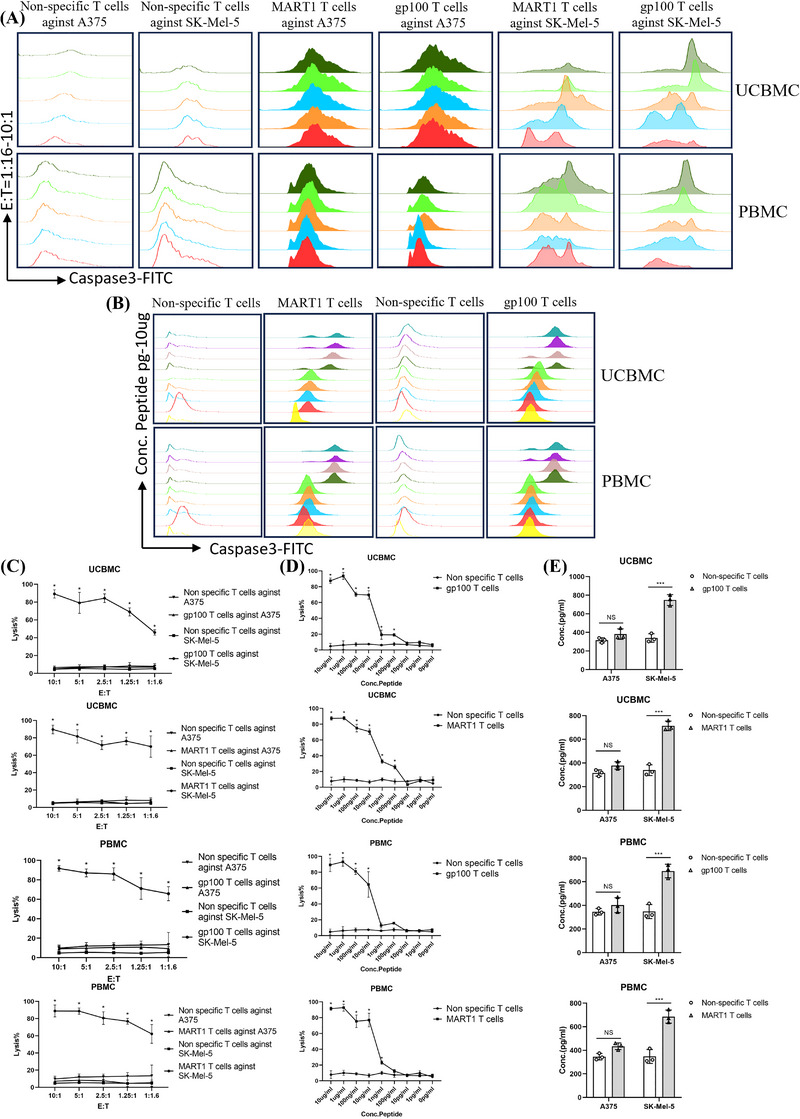
Functional avidity of MART1 and gp100 antigen‐specific CD8^+^ T cells from UCBMCs and PBMCs. (A) Flow cytometry analysis demonstrates that melanoma cell lines are effectively killed by UCBMCs and PBMCs antigen‐specific CD8^+^ T cells, as indicated by Caspase 3 detection, compared to non‐specific T cells. (B) This technique reveals the cytotoxicity of antigen‐specific CD8^+^ T cells targeting T2 cells presenting MART1 and gp100 antigen peptides, including both UCBMCs‐ and PBMCs‐derived non‐specific T cells and antigen‐specific CD8^+^ T cells on T2 cells loaded with various peptide concentrations. (C) The statistical analysis of non‐specific T cells and MART1 and gp100 antigen‐specific CD8^+^ T cells derived from UCBMCs and PBMCs is presented in three different effector‐to‐target cell ratios: E:T = 10:1, 5:1, 2.5:1, 1.25:1 and 1:1.6. The data for MART1 and gp100 antigen‐specific CD8^+^ T cells are statistically significant. (D) For specific lysis at an E:T ratio of 10:1, flow cytometry verified the apoptosis of T2 cells loaded with varying MART1 and gp100 peptide concentrations (10 µg to 0 pg) using the Caspase 3 apoptosis index. (E) Data show that co‐culture antigen‐specific CD8^+^ T cells with target cells promotes IFN‐γ release. MART1 and gp100 antigen‐specific CD8^+^ T cells release significantly more IFN‐γ compared to non‐specific T cells derived from UCBMCs and PBMCs, except in the A375 cell group, where no significant difference was observed. *N* = 3; ***p <0.05, ***p < 0.001.

The cytotoxicity of antigen‐specific CD8^+^ T cells from UCBMCs and PBMCs against melanoma cell lines Sk‐Mel‐5 and A375 was evaluated using flow cytometry assays, specifically measuring caspase 3‐induced apoptosis. Compared to the lysis caused by non‐specific T cells from UCBMCs and PBMCs, MART1 and gp100 antigen‐specific CD8^+^ T cells showed statistically significant lysis of Sk‐Mel‐5 cells but not of A375 melanoma cells (Figure 2A,C). The apoptosis index via caspase 3 was assessed to determine cell lysis of T2 cells, which were titrated with MART1 and gp100 peptides. MART1 antigen‐specific CD8^+^ T cells from UCBMC exhibited a significant cytotoxic effect on T2 cells loaded with MART1 peptide concentrations ≥100 pg/mL, compared to non‐specific T cells from UCBMCs. Similar results were observed for gp100 antigen‐specific CD8^+^ T cells from UCBMCs, which effectively lysed T2 cells with gp100 peptide titers ≥100 pg/mL. Likewise, MART1‐specific CD8^+^ T cells derived from PBMCs lysed T2 cells loaded with ≥1 ng/mL MART1 peptide, while gp100‐specific CD8^+^ T cells from PBMCs demonstrated lysis of T2 cells at gp100 peptide concentrations ≥10 ng/mL (Figure 2B,D).

### 3.6 IFN‐γ secretion of antigen‐specific CD8^+^ T cells

IFN‐γ plays an essential role in inhibiting tumour proliferation, inducing apoptosis, preventing metastasis, enhancing immunogenicity, and regulating T‐cell infiltration in tumours, thus serving as a critical marker for T‐cell function. Compared to non‐specific T cells from UCBMCs, MART1‐expressing SK‐Mel‐5 cells significantly stimulated IFN‐γ secretion from MART1‐specific CD8^+^ T cells isolated from UCBMCs, whereas A375 cells did not present this stimulation. Similarly, gp100 antigen‐specific CD8^+^ T cells from both UCBMCs and PBMCs exhibited enhanced IFN‐γ secretion when stimulated by SK‐Mel‐5 cells but not by A375 cells, which do not express the gp100 antigen (Figure 2E).

### 3.7 Antigen‐specific CD8^+^ T cells kill tumours in vivo

**FIGURE 3:**
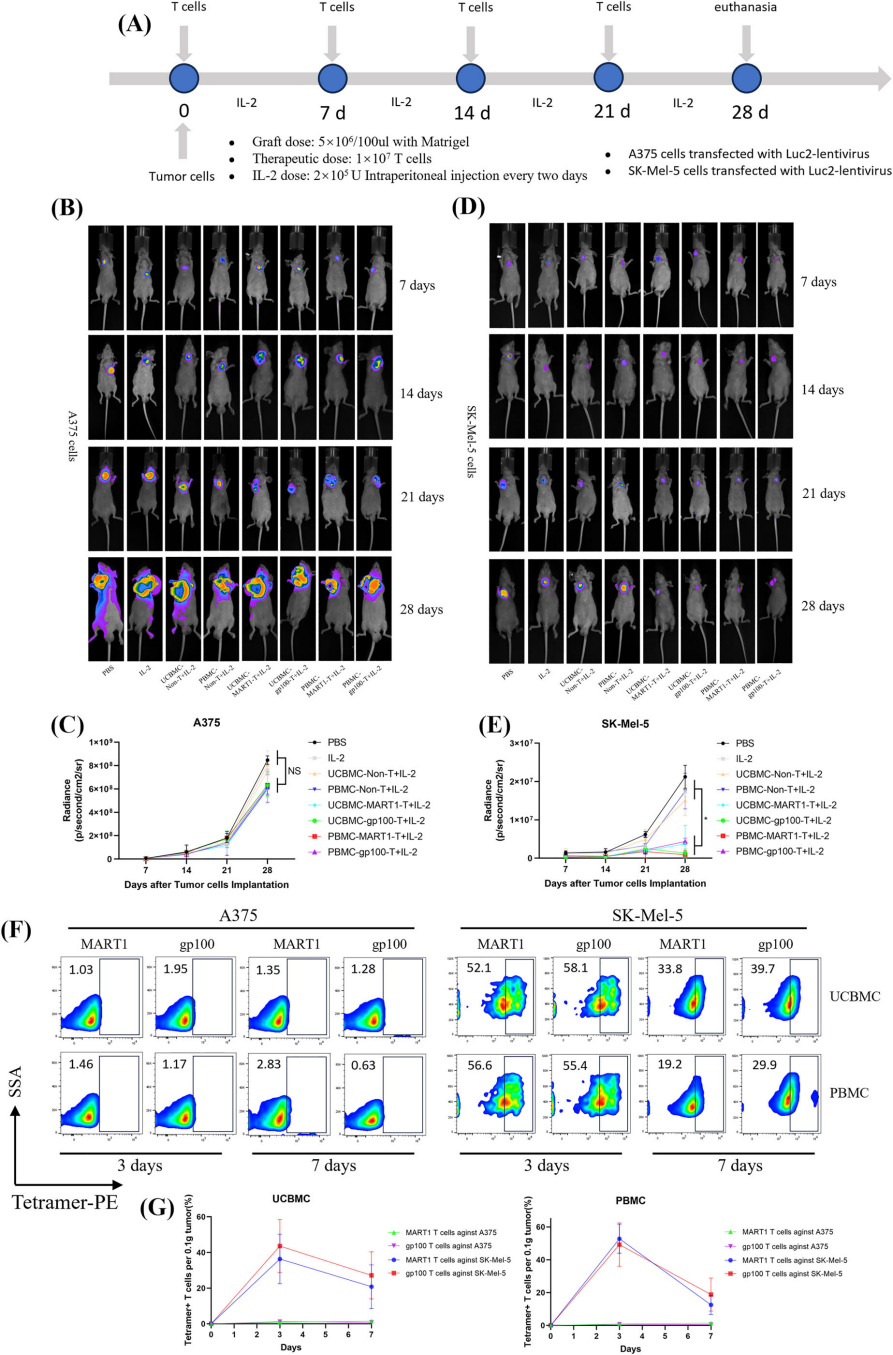
Experiment of anti‐tumour assessment by treatment with MART1 and gp100 antigen‐specific T cells in vivo. (A) Diagram of antigen‐specific T cells targeting melanoma in vivo. (B) Live imaging of nude mouse tumours on Days 7, 14, 21 and 28 post‐inoculation with A375 cells, following activation of the luciferase reporter gene by the substrate. (C) Statistical analysis of fluorescence intensity in nude mice on Days 7, 14, 21 and 28 after transplantation with A375 cells. (D) Live imaging of nude mouse tumours on Days 7, 14, 21 and 28 after inoculation with SK‐Mel‐5 cells, following substrate‐induced activation of the luciferase reporter gene. (E) Statistical maps depicting fluorescence intensity on Days 7, 14, 21 and 28 post‐inoculation of SK‐MEL‐5 in nude mice. *N* = 3, NS >0.05; *p <0.05. (F–G) A375 and Sk‐Mel‐5 tumour cells were engrafted in animal models. After adoptive transfer of antigen‐specific CD8⁺ T cells, lymphocytes were isolated from tumour tissues on Days 3 and 7 post‐injection. Flow cytometry with tetramer staining showed minimal infiltration of antigen‐specific CD8⁺ T cells in A375 tumours (0.5%–3.0%), while Sk‐Mel‐5 tumours exhibited significantly higher infiltration. In Sk‐Mel‐5 tumours, antigen‐specific CD8⁺ T cells rapidly accumulated at the third day (40% for UCBMCs‐derived and 50% for PBMCs‐derived), but declined 25% and 17%, respectively, at the seventh day. The data represent the mean ± standard deviation of three independent experiments.

The luciferase reporter gene, luc2, was transfected into melanoma cell lines SK‐Mel‐5 and A375, resulting in stable expression cell lines luc2‐A375 and luc2‐SK‐Mel‐5. This was confirmed through GFP fluorescence, satisfying the requirements for tumour imaging experiments in vivo (Figure S1I). The cytotoxic effects of antigen‐specific CD8^+^ T cells in vivo were assessed at 7, 14, 21 and 28 days post‐transplantation of luc2‐A375 cells, following treatments with PBS injection group, IL‐2 injection group and non‐specific T cells derived from UCBMCs and PBMCs injection group. No significant differences were observed between injection groups of MART1 and gp100 antigen‐specific CD8^+^ T cells derived from UCBMCs and PBMCs (Figure 3B,C). Comparisons were made at 7, 14 and 21 days after luc2‐SK‐Mel‐5 transplantation among the PBS injection group, IL‐2 injection group, and non‐specific T cells derived from UCBMCs and PBMCs injection groups. No significant differences were found among MART1 antigen‐specific CD8^+^ T cells from the UCBMCs injection group, gp100 antigen‐specific CD8^+^ T cells from the UCBMCs injection group, MART1 antigen‐specific CD8^+^ T cells from the PBMCs injection group, and gp100 antigen‐specific CD8^+^ T cells from the PBMCs injection group. However, on Day 28, there was a statistically significant difference observed among the antigen‐specific CD8^+^ T‐cell group compared to the PBS injection group, IL‐2 injection group, and non‐specific T cells derived from UCBMCs and PBMCs injection group (Figure 3D,E).

### 3.8 Cell kinetics characteristics of antigen‐specific CD8^+^ T cells

A375 and Sk‐Mel‐5 tumour cells were successfully engrafted in animal models, leading to the establishment of tumours. Following the adoptive transfer of antigen‐specific CD8⁺ T cells, lymphocytes were isolated from tumour tissues on post‐injection Days 3 and 7. Flow cytometry analysis using tetramer staining demonstrated minimal intratumoural infiltration of antigen‐specific CD8⁺ T cells in the A375 group, with frequencies ranging from 0.5% to 3.0% on both Days 3 and 7. In contrast, the Sk‐Mel‐5 group exhibited significantly enhanced infiltration, as shown in Figure 3F. Notably, antigen‐specific CD8⁺ T cells rapidly accumulated in Sk‐Mel‐5 tumours by Day 3, with mean frequencies of approximately 40% for UCBMC‐derived cells and 50% for PBMC‐derived cells. However, infiltration declined by Day 7 to approximately 25% for UCBMC‐derived cells and 17% for PBMC‐derived cells (Figure 3G). This temporal pattern suggests that UCBMC‐derived antigen‐specific T cells exhibit superior persistence within tumour‐infiltrating populations.

### 3.9 Characterisation of MART1 and gp100 antigen‐specific T cells by scRNA‐seq

**FIGURE 4:**
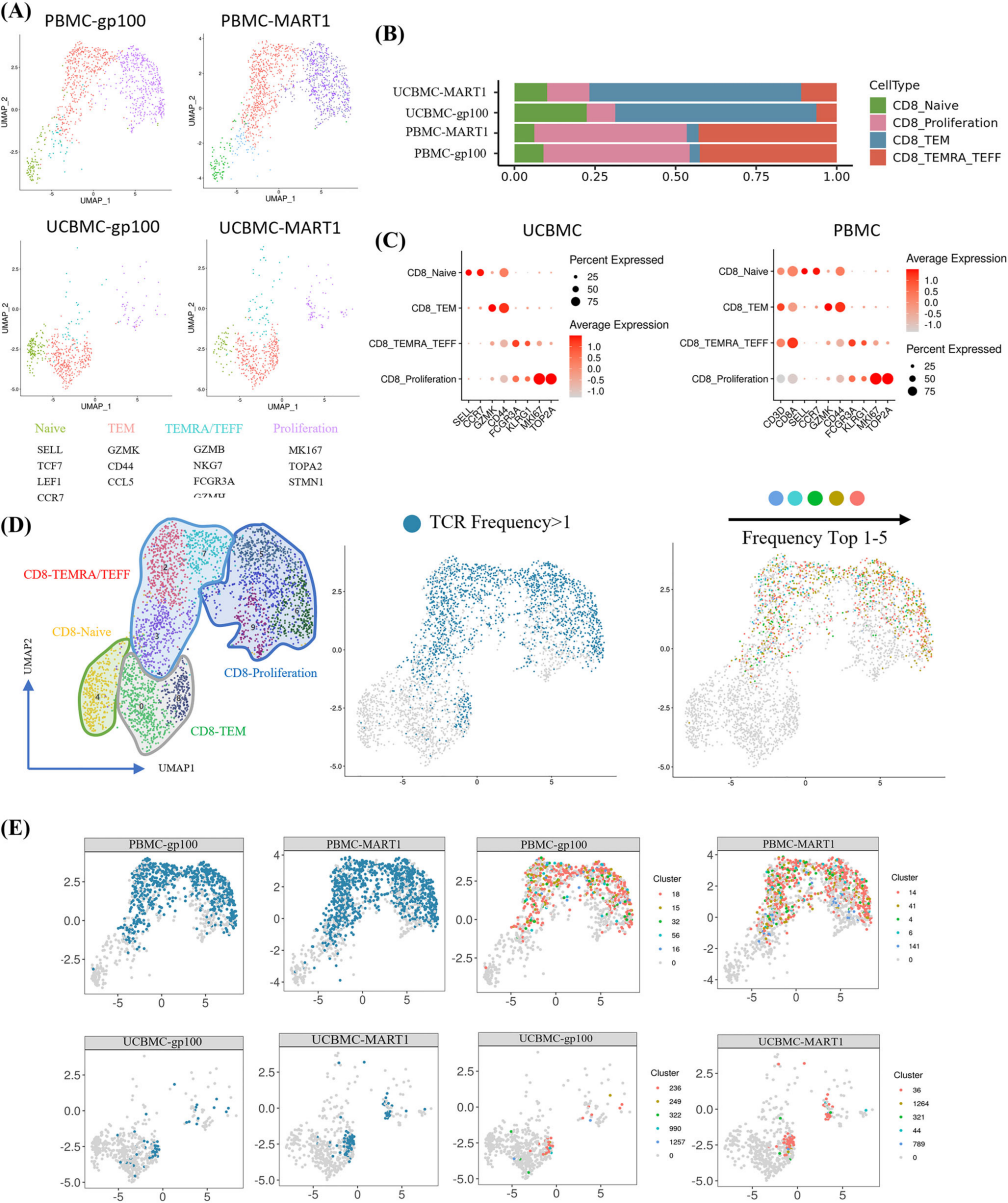
Single‐cell and TCR landscape of antigen‐specific CD8^+^ T cells of MART1 and gp100. (A) UMAP visualisation of the scRNA‐seq data from tetramer‐sorted MART1 and gp100 antigen‐specific CD8^+^ T cells is presented. The identified cell clusters (*n* = 10) are depicted in distinct colours, with cluster‐specific genes listed adjacent to each cluster ID. (B) The proportion of each cell subpopulation in antigen‐specific T cells from different sources is shown. (C) A bubble diagram illustrates marker genes for each T‐cell subtype. The colour scale indicates the average normalised expression of marker genes in each subtype, while dot size represents the percentage of cells within each cluster that express the marker gene. (D) UMAP visualisation of the TCR‐seq from tetramer‐sorted antigen‐specific CD8^+^ T cells is displayed on the left. In the middle, UMAP visualisation with TCR clonotype expansion information (clonotype frequency >1) is shown. On the right, the top five most frequent TCR clonotypes are highlighted for MART1 and gp100. (E) UMAP visualisation shows TCR clonotype expansion (clonotype frequency >1) in MART1 and gp100 antigen‐specific T cells from UCBMCs and PBMCs. The five TCR clonotypes with the highest expression in MART1 and gp100 antigen‐specific T cells from these sources are also expanded.

Uniform Manifold Approximation and Projection (umap) and principal components analysis (PCA) algorithms were used for dimensionality reduction and information presentation. Through scRNA‐seq quality control analysis of UCBMC and PBMC antigen‐specific CD8^+^ T cells, we found that the nCounts, nFeatures and mitochondrial values for all samples were within a reasonable range, indicating good overall correlation between the samples. This provided a reference for further screening of low‐quality cells. We successfully generated high‐quality scRNA‐seq data from antigen‐specific CD8^+^ T cells with potential specificity to the MART1 and gp100 epitopes. Based on transcriptomic profiles, the tumour antigen‐specific CD8^+^ T cells were divided into four cell subsets, and marker genes for each cell subset were identified. Cluster 4 was annotated as naïve CD8^+^ T cells, characterised by high expression of SELL, TCF7, LEF1 and CCR7. Clusters 2, 3 and 7 were annotated as TEMRA/TEFF CD8^+^ T cells, or effector T cells, based on markers such as GZMB, NKG7, CST7, FCGR3A and GZMH. Cluster 0 was identified as TEM CD8^+^ T cells, or effector‐memory T cells, characterised by high expression of GZMK, CD44 and CCL5. Clusters 1, 5, 6 and 9, with high expression of MKI67, TOP2A and STMN1, were identified as proliferation CD8^+^ T cells (Figure 4A, Figure S2A–D). Among MART1 antigen‐specific CD8^+^ T cells derived from UCBMCs, 10.13% were naive cells, 13.12% were proliferation cells, 65.67% were TEM cells and 11.07% were TEMRA/TEFF cells. For gp100 antigen‐specific CD8^+^ T cells from UCBMCs, these proportions were 22.46% naive cells, 8.77% proliferation cells, 62.46% TEM cells and 6.32% TEMRA/TEFF cells. Among MART1 antigen‐specific CD8^+^ T cells derived from PBMCs, the proportions were 6.13% naive cells, 47.33% proliferation cells, 3.68% TEM cells and 42.86% TEMRA/TEFF cells. For gp100 antigen‐specific CD8^+^ T cells from PBMCs, 9.01% were naive cells, 45.35% were proliferation cells, 3.16% were TEM cells and 42.47% were TEMRA/TEFF cells (Figure 4B). Subsequently, we functionally characterised MART1 and gp100 antigen‐specific CD8^+^ T cells using gene panels associated with naive, proliferation, TEM and TEMRA/TEFF CD8^+^ T‐cell subsets. The results indicated that naive CD8^+^ T‐cell subsets were relatively more naïve, whereas TEMRA/TEFF CD8^+^ T‐cell subsets exhibited higher activation and cytotoxicity, and proliferative CD8^+^ T cells were indeed more proliferative. Furthermore, TEM CD8^+^ T cells demonstrated higher cytotoxicity and slight proliferation compared to naïve cells, aligning with observations that effector CD8^+^ T cells had greater cytotoxicity and clonal expansion (Figure 4C). We also analysed scRNA‐seq data with SCENIC to identify potential co‐expression modules and associated cis‐regulatory elements. The most overrepresented motifs included those for oncoproteins FOS and E2F7, as well as the interferon‐dependent transcription factor STAT1, all enriched in proliferating cells (Figure S3A). Additionally, we employed CellChat to conduct intercellular interaction analysis, which maps ligand–receptor pair expressions between T‐cell subsets, allowing inference of potential intercellular interactions. The association of CCR1 and KIR2DL3 genes in various T‐cell subsets is primarily strengthened through HLA class molecules (Figure S3B).

### 3.10 Subsets of T cells and TCR immune repertoire analysis

**FIGURE 5:**
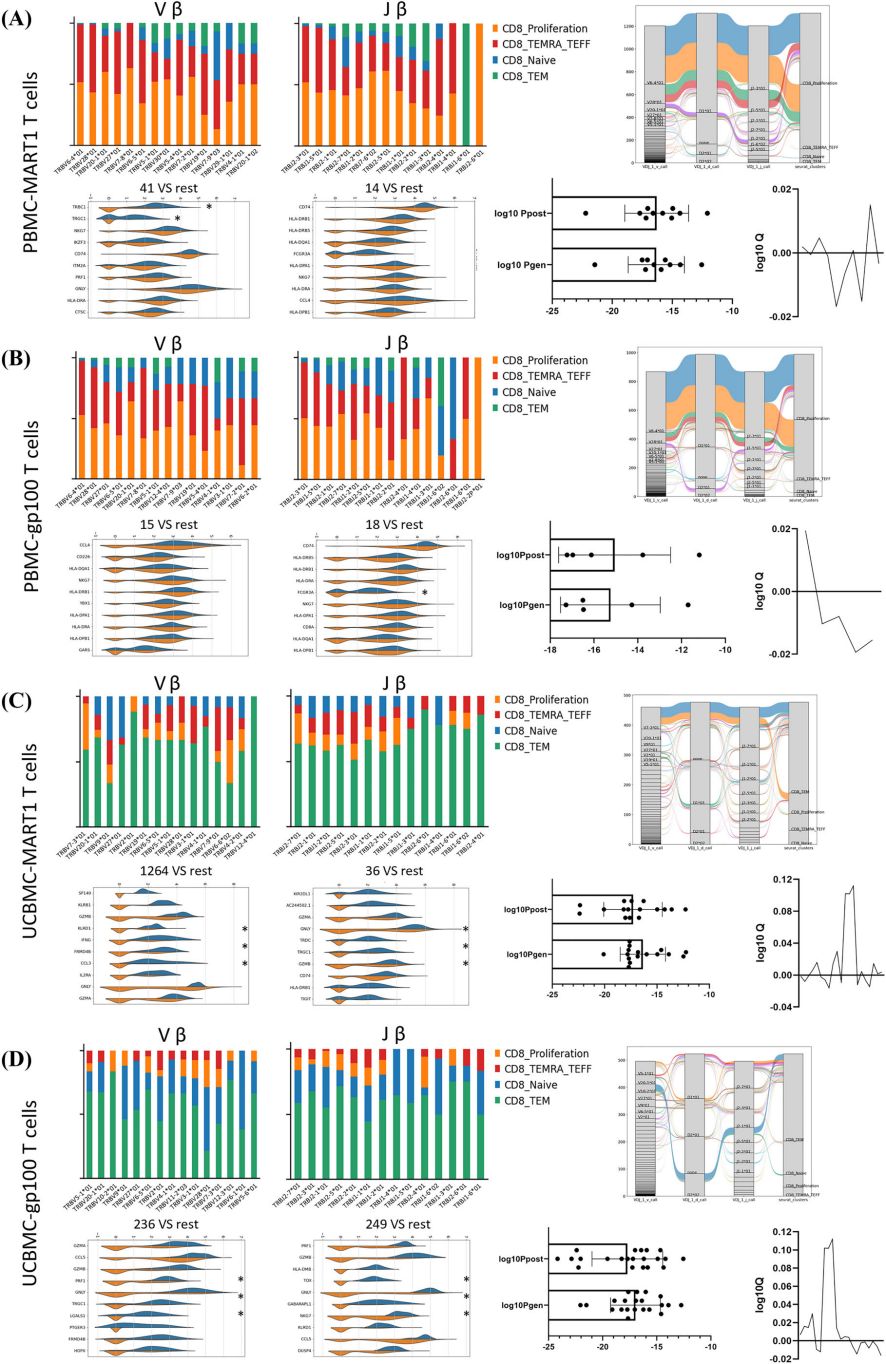
Expression sequence of Vα and Vβ in TCR region. (A) Sankey diagram illustrates the clonality and distribution of MART1 antigen‐specific CD8^+^ T cells αβ V(D)J from PBMC. TRBV4‐6*01 and TRBV28*01 are the top two expressed Vβ. Violin plots are used for clustering annotation with key gene markers, showing that TRBC1, TRGC1 and FCGR3A are significantly more prevalent than other genes. TCR repertoire analysis using the SOS algorithm revealed limited clonal diversity in antigen‐specific CD8⁺ T cells. For MART‐1‐specific T cells derived from PBMCs, log₁₀*Q* values ranged from −0.01678 to .015046 (mean = −0.00207). (B) The clonality and distribution of gp100 antigen‐specific CD8^+^ T cells αβ V(D)J from PBMC revealed TRBV4‐6*01 and TRBV28*01 as the top two Vβ expressions. Violin plots used for clustering annotations demonstrated that FCGR3A was significantly expressed compared to other genes. Similarly, gp100‐specific T cells exhibited log₁₀*Q* values between −0.01938 and 0.019451 (mean = −0.00674). (C) For MART1 antigen‐specific CD8^+^ T cells αβ V(D)J from UCBMCs; the top two Vβ expressions are TRBV7‐301 and TRBV20‐1*01. TRBJ2‐701 and TRBJ2‐1*01 are the top two expressions of Jβ. The violin plots show that GZMA and other identified genes are significantly more active than other genes. TCR repertoire analysis using the SOS algorithm revealed log₁₀*Q* values for UCBMC‐derived MART‐1‐specific T cells ranging from −0.0101 to 0.145438 (mean = 0.022771). (D) The gp100 antigen‐specific CD8^+^ T cells αβ V(D)J from UCBMC show that TRBV5‐1*01 and TRBV20‐1*01 are the top two Vβ. Violin plots for cluster annotations indicate that GZMB is significantly expressed relative to other genes. Similarly, gp100‐specific T cells exhibit log₁₀*Q* values between −0.01618 and .112133 (mean = 0.015743). *p < .05, ***p < .001.

**FIGURE 6:**
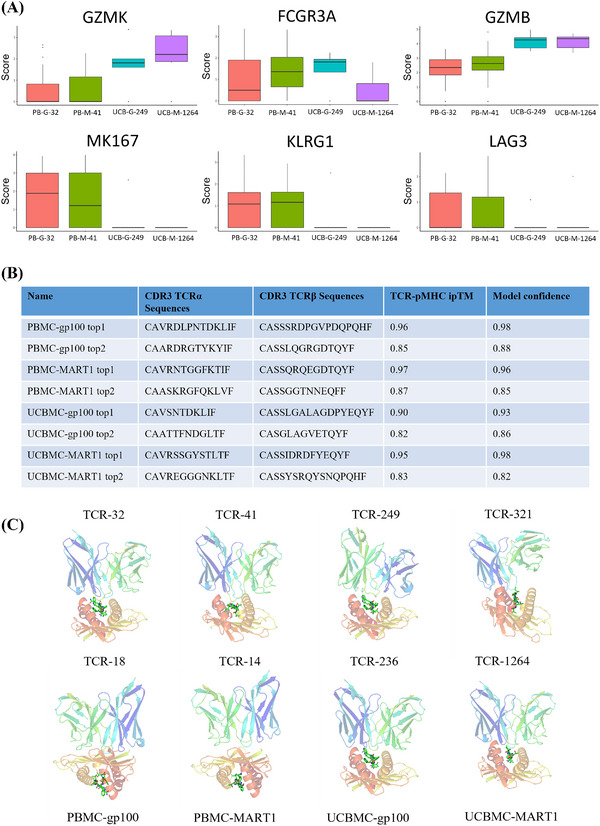
Expression of immune‐related genes and molecular docking of candidate TCRs. (A) The boxplot illustrates the expression levels of immune‐related genes in CD8^+^ T cells with the highest number of TCR clones. In these boxplots, the edges of the boxes represent the first and third quartiles, while the line inside each box indicates the median. (B) A summary is provided of the predicted TCR structure of eight HLA‐A2‐restricted MART1 and gp100 peptides. (C) The simulated docking structure of the pMHC–TCR complex is presented for five candidate TCRs with MART1 and gp100. In depiction, the top section shows the TCR structure, the bottom section displays the HLA‐A2 presenting the MART1 and gp100 peptides, and the central section depicts the peptide structure.

TCR‐seq is a powerful tool for identifying TCR clonotypes that target tumour antigens and can provide complete pairing information for the α and β chains of TCRs. However, most antigen‐specific TCRs exhibit low affinity for tumour‐associated pMHCs, leading to weak activation of the T cells to which they bind. Therefore, screening for TCRs with the appropriate affinity for tumour antigens is crucial. The results demonstrated that TCR clonotype expansion (where clonotype frequency exceeds one) accounted for 64.91%–69.05% within PBMCs, with the top five clonotypes collectively representing 58.43%–60.39% of PBMCs, predominantly identified in CD8^+^ T cells in the TEMRA/TEFF and proliferative states. In UCBMCs, TCR clonotype expansion accounted for 9.65%–18.01%, with the top five clonotypes representing 6.37%–12.52%, mostly observed in CD8^+^ T cells in the TEM state. Considering overall TCR clonotype expansion, eight clonotypes (TCR‐32, TCR‐18, TCR‐41, TCR‐14, TCR‐249, TCR‐236, TCR‐321 and TCR‐1264) were selected for further analysis and validation. TCR‐32, TCR‐41, TCR‐249 and TCR‐1264 exhibited the highest clonotype frequencies (Figure 4D,E). Separate V(D)J pairings of α and β chains revealed significantly higher frequencies of TRAV6‐4*01, TRAJ2‐3*01, TRBV28*01 and TRBJ1‐5*01 in MART1 and gp100 antigen‐specific CD8^+^ T cells from PBMC compared to other TCR sequences. Highly clonal TCRs were primarily found in CD8‐proliferation and TEMRA/TEFF CD8^+^ T cells. Among the top 15 most clonally expanded antigen‐specific CD8⁺ T cells, immune‐related gene expression profiles showed no significant differences compared to lower clonality CD8⁺ T cells derived from PBMCs, except for elevated expression of TRBC1, TRGC1 and FCGR3A. TCR repertoire analysis using the SOS algorithm revealed limited clonal diversity in antigen‐specific CD8⁺ T cells. For MART‐1‐specific T cells derived from PBMC, log₁₀Q values ranged from −.01678 to .015046 (mean = −0.00207). Similarly, gp100‐specific T cells exhibited log₁₀*Q* values between −0.01938 and .019451 (mean = −0.00674), indicating consistently constrained diversity profiles (Figure 5A,B; Figure S4A,C). In MART1 antigen‐specific CD8^+^ T cells derived from UCBMCs, TRAV29*01, TRAJ23*01, TRBV7‐3*01 and TRBJ2‐7*01 were most frequently used, while TRBV13‐1*01, TRAJ45*01, TRBV5‐1*01 and TRBJ2‐7*01 were prevalent among gp100‐specific CD8^+^ T cells. Immune‐related gene expression profiles significantly differed between the top 15 most clonally expanded antigen‐specific CD8⁺ T cells and lower clonality CD8⁺ T cells derived from UCBMCs. This differential expression suggests that combining alternative immunotherapies with UCBMC‐derived antigen‐specific CD8⁺ T cells may enhance therapeutic efficacy. TCR diversity analysis using the SOS algorithm revealed log₁₀*Q* values for UCBMCs‐derived MART‐1‐specific T cells ranging from −0.0101 to 0.145438 (mean = 0.022771). Similarly, gp100‐specific T cells exhibited log₁₀Q values between −.01618 and .112133 (mean = 0.015743) (Figure 5C,D, Figure S4B,D). This suggests that antigen‐specific CD8^+^ T cells derived from UCBMC could potentially enhance therapeutic outcomes when combined with other immunotherapies. Additionally, effector CD8^+^ T‐cell marker genes (GZMB, FCGR3A) were highly expressed in TCR‐249 and TCR‐1264 clonotypes, proliferation marker genes (MKI67) in TCR‐32 and TCR‐41 clonotypes, and exhaustion markers (GZMK, KLRG1, LAG3) also in TCR‐32 and TCR‐41 clonotypes (Figure 6A). Overall, these findings propose that CD8^+^ T cells with the eight selected TCR clonotypes may exhibit a high tumour‐killing effect by targeting MART1 and gp100 epitopes.

### 3.11 Structure docking

We employed a local protein docking prediction tool, TCRmodel2, to evaluate the affinity between pMHC and TCR molecules. The MART1 and gp100 epitopes, presented by HLA‐A2, were examined for binding stability with eight candidate TCRs, and five models were generated for each TCR‐pMHC complex. The ipTM score, ranging from 0 to 1, assessed the topological accuracy of the interface between TCR and pMHC, with higher values indicating greater feasibility. A model confidence score, also ranging from 0 to 1, represented a weighted summation of pTM and ipTM scores, where values equal to or greater than 0.85 were considered likely to be reliable. Most of the tested models exhibited a model confidence score of 0.85 or higher, suggesting that the top 1–2 MART1 and gp100 TCRs demonstrated superior binding stability (Figure 6B,C).

## 4 DISCUSSION

ETC has demonstrated clinical efficacy in patients with refractory metastatic melanoma and other solid malignant tumours.^33^ It offers enhanced flexibility, a quicker transition from antigen discovery to T‐cell therapy, and can potentially shorten treatment duration, thereby significantly improving the survival rates of patients with late‐stage malignant melanoma.^34^ Currently, antigen‐specific CD8^+^ T cells utilised in ETC therapy are sourced from PBMCs. We applied the foundational principles of ETC therapy to select appropriate HLA‐A2 PBMCs and UCBMCs to induce antigen‐specific CD8^+^ T cells capable of recognising tumour antigen targets. Although previous studies successfully induced antigen‐specific CD8^+^ T cells from PBMCs with sufficient affinity, our findings indicate that UCBMCs can also produce antigen‐specific CD8^+^ T cells.^18^, ^35^, ^36^, ^37^

In this study, we demonstrated that antigen‐specific CD8^+^ T cells, induced and mass‐produced via autologous dendritic cells loaded with MART1 and gp100 peptides, from both UCBMCs and PBMCs, exhibit sufficient functional affinity. Compared with non‐antigen‐specific T cells from UCBMCs and PBMCs, antigen‐specific CD8^+^ T cells could lyse T2 cells loaded with low concentrations of MART1 and gp100 peptides, as well as the SK‐Mel‐5 tumour cell line expressing MART1 and gp100, but had no evident cytotoxic effects on the A375 tumour cell line lacking MART1 and gp100 expression. These findings strongly suggest that antigen‐specific CD8^+^ T cells derived from UCBMCs and PBMCs have a strong affinity and can efficiently lyse tumour cells. Furthermore, we performed comprehensive repertoire sequencing of MART1 and gp100 antigen‐specific TCRs, assessing both cell phenotype and T‐cell clonal expansion. TCR candidates with high specificity and cytotoxic potential for MART1 and gp100 antigens were systematically screened and validated. These results highlight the potential for developing personalised TCR‐T‐cell therapy and cellular immunotherapy for melanoma.

Cell transplantation, similar to organ transplantation, presents challenges such as HLA mismatch, sourcing difficulties and transplantation delays. UCB serves as an excellent source of immune cells due to its ease of collection and noninvasive nature, making it an essential resource in immune cell transplantation for nearly a century.^38^, ^39^ Compared with PB transplantation, the biggest advantage of UCB transplantation is that the risk of GVHD is very low, and its immune cells are not stimulated by the antigenic determinant because the cells are in a relatively naive stage. This immune immaturity reduces the allogeneic response potential of lymphocytes and is key to reducing the severity of GVHD.^40^

Immune cells derived from UCBMCs are more compatible with transplantation, with T cells exhibiting greater proliferation and persistence capabilities post‐maturation than T cells from PBMC, a characteristic attributed to a higher prevalence of naive T cells. Our study compared the functional characteristics of antigen‐specific CD8^+^ T cells from UCBMCs and PBMCs, induced by HLA‐A2‐restricted MART1 and gp100‐peptide‐loaded DC stimulation. While no significant differences in proliferative or anti‐tumour abilities were observed between the cell groups, antigen‐specific CD8^+^ T cells derived from UCBMCs showed greater proliferation and cytotoxic potential than those from PBMCs. Previous studies demonstrated that amplified antigen‐specific CD8^+^ T cells derived from UCBMCs are polyclonal with functional and activated phenotypes. Another study reported antigen‐specific CD8^+^ T cells derived from UCBMCs primarily exhibit naive phenotypes with high proliferation potential.^41^ Although our findings are inconsistent with these reports, they support UCBMCs potential as therapeutic material for tumour cell transplantation without a definitive answer on its advantage over PBMC.

The clinical translation of UCB‐derived immune cell faces significant theoretical and technical hurdles. Key challenges include: (i) developing methods for the efficient expansion and isolation of UCB immune cells to achieve therapeutic doses for transplantation and adoptive therapy; (ii) assessing and mitigating safety concerns and immunogenicity arising from HLA mismatches; and (iii) identifying immune cell populations with enhanced tumour‐targeting capability while minimising autoreactivity.^42^ Overcoming these barriers requires substantial research breakthroughs to enable the broader and more effective application of UCB immune cell transplantation for diverse diseases.

A critical obstacle in adoptive therapy using UCB is generating sufficient quantities of T cells. Current data indicate that 80–120 mL of UCB typically yields 1.0 × 10⁹ UCBMCs, with T cells comprising 35%–40%. Approximately 70% of these T cells exhibit a naïve phenotype.^43^ This composition inherently limits the yield of UCB‐derived CD8⁺ T cells available for therapy. Furthermore, our research demonstrates that UCB‐derived immune cells generate significantly fewer antigen‐specific CD8⁺ T cells upon tumour antigen stimulation compared to peripheral blood‐derived counterparts. In vitro expansion of sorted UCB‐derived CD8⁺ T cells using IL‐7/IL‐21 cytokines and feeder cells achieved expansion indices of 600–1000. While other studies report UCB T‐cell expansions exceeding 2500‐fold, our lower efficiency likely results from factors including: (i) an estimated 30% loss of cell viability due to cryopreservation/thawing before culture, and (ii) the use of autologous serum. Although autologous serum offers potential safety advantages over allogeneic serum or fetal bovine serum (FBS), serum‐free media are well‐documented to achieve 20%–40% lower expansion efficiencies compared to serum‐supplemented systems.^44^, ^45^, ^46^, ^47^, ^48^, ^49^ Consequently, achieving an appropriate balance between expansion efficiency and safety is essential and must be guided by clinical requirements. Importantly, excessive expansion (>2000‐fold) risks inducing T‐cell exhaustion, potentially compromising in vivo persistence and long‐term efficacy. Notably, our animal studies indicate that UCB‐derived antigen‐specific T cells exhibit enhanced persistence and superior resistance to exhaustion. Therefore, therapeutic cell quantity cannot be considered in isolation; it necessitates careful evaluation of source‐specific functional characteristics and survival kinetics. Further research using robust experimental data is imperative to optimise UCB‐derived T‐cell expansion protocols for clinical application.

Despite global investigations into UCBMCs clinical applications, limited knowledge exists on its cellular and molecular characteristics. We continue to lack a comprehensive understanding of lymphocyte‐affecting reconstruction following UCBMC transplantation. However, advanced single‐cell transcriptomics now enable gene expression analysis at the cellular level, allowing in‐depth exploration of inter‐cell heterogeneity and functional correlations to improve understanding of UCBMC immune cell transplantation mechanisms. ScRNA‐seq studies have offered new insights into UCBMC immune cell composition and disease‐associated abnormalities, revealing that nucleated cell functions and mechanisms in anti‐cancer therapy remain underexplored. These cell types hold significant importance in clinical medicine and require further functional characterisation.^50^

We induced and cultured two types of antigen‐specific CD8^+^ T cells from distinct sources in vitro, subsequently amplifying them for anti‐tumour functional analysis. However, detailed examination of cell subset composition, transcriptional regulation and receptor–ligand interactions remains necessary. Previous studies revealed CD8^+^ T cells with different molecular markers, identifying naive T cells expressing CCR7, TCF7, LEF1 and SELL, alongside cytotoxic T cells marked by PRF1, GZMA, GZMB and NKG7. A separate study defined naive T cells by CCR7, LEF1, IL7R and TCF7 expression, while identifying cytotoxic T cells using CX3CR1, PRF1, KLRG1 and FGFBP2 expression. Findings from a third study concurred, observing naive T cells expressing CCR7, IL7R, LEF1 and TCF7, with ‘cytotoxic’ T cells defined by FCGR3A, KLRG1, PRF1 and GZMB expression.^51^, ^52^, ^53^ Our study determined variance in antigen‐specific CD8^+^ T‐cell proportions between UCBMCs and PBMCs relative to anti‐tumour efficacy. The proportions of proliferative cells, effector cells and naive and effect‐memory cells in PBMC‐derived antigen‐specific CD8^+^ T cells were 45%–48%, 40%–45% and 10%–15%, respectively, whereas UCBMC‐derived effector‐memory cells amass 60%–65%, with proliferative and effector cells at 15%–25%, and naive cells at 10%–20%. Our research in animal models demonstrated that tumour tissues receiving antigen‐specific CD8⁺ T‐cell infusions exhibited comparable levels of tumour‐infiltrating antigen‐specific CD8⁺ T cells derived from UCB and PB by Day 7 post‐administration. However, limited longitudinal data suggest that memory T‐cell persistence and expansion efficiency in vivo may be compromised by tumour microenvironment (TME)‐mediated suppression, potentially resulting in lower long‐term engraftment than anticipated. Additionally, prolonged exposure of UCB‐derived T cells to tumour antigens may promote tolerance induction, characterised by functional anergy or apoptosis, thereby accelerating immune escape.^54^ Concurrently, chronic antigen stimulation or inflammatory factors can drive memory T‐cell exhaustion, diminishing proliferative capacity and promoting terminal differentiation into effector phenotypes. This exhaustion process shortens the therapeutic window for sustained anti‐tumour activity.^55^

ScRNA‐seq enhances our understanding of subsets of antigen‐specific CD8^+^ T cells. During cell therapy, TCR affinity remains pivotal; identifying tumour‐reactive TCRs with appropriate affinity is essential for therapeutic success. We enriched MART1 and gp100 antigen‐specific CD8^+^ T cells, generating high‐quality transcriptome and TCR data. MART1 and gp100 antigen‐specific CD8^+^ T cells transcriptome and TCR repertoire data offer a critical resource for melanoma‐targeted T‐cell screening with high anti‐tumour responses. TCR sequencing analysis of antigen‐specific T cells derived from PB and UCB revealed limited overlap in the CDR3 region—the central structural element responsible for peptide–MHC recognition—among different T cells targeting the same antigen. Specifically, only approximately 30%–40% of the top 15 CDR3β sequences were shared. This limited sequence convergence arises from the structural basis of TCR cross‐reactivity: while anchor residues within the antigenic peptide dictate core TCR–CDR3 interactions, non‐anchor residues accommodate considerable substitution. The theoretical diversity of possible antigenic peptides exceeds 10^15^–10^16^ unique sequences, vastly outstripping the estimated less than 10⁸ distinct TCR sequences within an individual. Furthermore, thymic negative selection eliminates T cells bearing high‐affinity self‐reactive TCRs, resulting in a peripheral TCR repertoire characterised predominantly by low‐to‐moderate affinity for any given antigen. Crucially, the absence of somatic hypermutation in TCR genes prevents rapid diversification of CDR3 regions in response to antigenic challenge. Consequently, T‐cell responses to both self and foreign antigens necessarily originate from this fixed, pre‐existing repertoire. These fundamental constraints dictate that single antigens are recognised by multiple distinct TCRs, necessitating inherent TCR cross‐reactivity.^56^, ^57^, ^58^

The inherent cross‐reactivity, rooted in the mechanism of MHC‐peptide recognition, presents a significant clinical challenge: the potential for TCRs to engage unexpected epitopes on healthy tissues, resulting in ‘off‐target toxicity’. Such unintended reactivity can provoke severe adverse events, including cytokine release syndrome (CRS), multi‐organ damage and immune effector cell‐associated neurotoxicity syndrome (ICANS). Although thymic selection removes T cells exhibiting strong autoreactivity, leaving a repertoire of predominantly medium‐to‐low affinity TCRs, clinical evidence indicates that off‐target toxicity remains a non‐negligible risk.

To mitigate this risk, our team will implement a comprehensive safety assessment strategy. First, we will employ advanced computational tools—including NetTCR, TCRex and the structural biology platform AlphaFold‐Multimer—to predict potential off‐target peptide–MHC interactions for candidate TCRs, prioritising experimental screening targets. Second, we will utilise validated humanised mouse models injected with UCB‐derived antigen‐specific CD8⁺ T cells. In these models, we will rigorously monitor for acute/chronic toxicity, histopathological alterations, cytokine profiles, evidence of off‐target tissue damage, and the emergence of CRS/ICANS. These preclinical studies aim to refine safety data and establish robust risk profiles before clinical translation.^59^, ^60^, ^61^, ^62^

In conclusion, T cells that deliver essential anti‐tumour activity exhibit notable heterogeneity and functional disparities. Evaluating their physiological and pathological effects poses a major precision medicine challenge. Previous high‐throughput sequencing provided limited insights due to sequencing depth constraints, leaving melanoma cell transplantation therapy research largely unexamined. In this study, we explored tumour antigen‐specific CD8^+^ T‐cell heterogeneity, statistically identifying significantly expressed genes, and clarified interactions between T cells and gene expression. Accurate TCR measurement remains critical for precision medicine, and our research on antigen‐specific CD8^+^ T cells derived from UCBMC and PBMC delivers essential data and relationships for future immune database development. With advancements in single‐cell sequencing, we now gain a comprehensive understanding of tumour‐associated CD8^+^ T cells, enabling precise identification and quantification of cancer‐associated T‐cell types, thus providing new avenues for cancer detection and treatment.

## AUTHOR CONTRIBUTIONS

**Hongwei Liu** and **Xiao Jiang**: designed the project. **Jiaji Liang** and **Xi He**: performed sample collection and conducted the experiments, and performed the bioinformatics analysis. **Zhiqin Dong**: assisted in the activation and cytotoxicity assay. **Jinqiang Lu** and **Shuxian Jiang**: analysed the data and wrote the manuscript with inputs from the other authors. **Hengyu Du**: guided the experiments. **Haoran Mao**: revised the manuscript. **Songtao Luo** and **Xifeng An**: assisted with sample collection.

## CONFLICT OF INTEREST STATEMENT

Patent applications have been filed on aspects of the described T‐cell culture method and candidate TCR sequences by Jiaji Liang, Xi He, Xiao Jiang and Hongwei Liu. The remaining authors declare no conflicts of interest.

## ETHICS STATEMENT

This study was approved by the Ethics Committee of the First Affiliated Hospital of Jinan University. (permit number: 20230309‐0004), and performed in accordance with all applicable guidelines and regulations.

## Supporting information



Supporting Information

## Data Availability

The raw data are available in the Genome Sequence Archive (GSA) under restricted access PRJCA033339 (https://ngdc.cncb.ac.cn). The raw sequencing data are available under controlled access, and the data should be used for research purposes only.
