# Supplementary material for: Umbilical cord blood‐derived cytotoxic T lymphocytes target melanoma via HLA‐A2‐restricted tumour antigens
Source: Clin Transl Med. 2025 Oct 15;15(10):e70444. doi: 10.1002/ctm2.70444 (PMC12521828; doi:10.1002/ctm2.70444)
Supplement: Supplementary file 1 — Supporting Information [file CTM2-15-e70444-s001.pptx]

## Slide 1
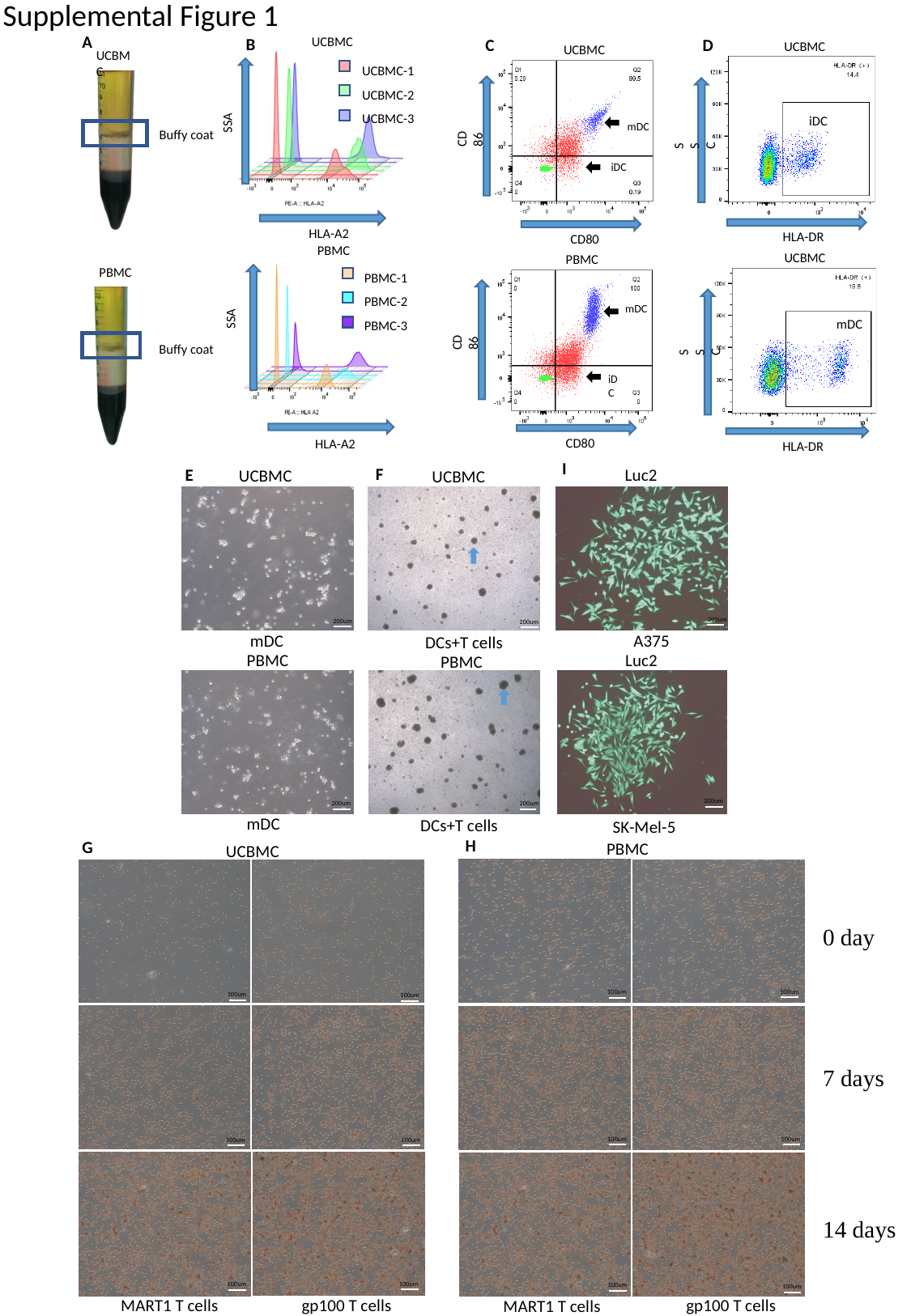

Supplemental Figure 1
A
UCBMC
Buffy coat
B
UCBMC
HLA-A2
UCBMC-1
UCBMC-2
UCBMC-3
SSA
C
UCBMC
CD86
CD80
mDC
iDC
D
UCBMC
iDC
HLA-DR
SSC
PBMC
HLA-A2
PBMC-1
PBMC-2
PBMC-3
SSA
UCBMC
mDC
HLA-DR
SSC
PBMC
Buffy coat
PBMC
CD86
CD80
mDC
iDC
I
F
UCBMC
DCs+T cells
200um
Luc2
E
UCBMC
200um
mDC
200um
A375
PBMC
200um
mDC
Luc2
PBMC
200um
DCs+T cells
200um
SK-Mel-5
H
G
PBMC
UCBMC
100um
100um
100um
100um
100um
100um
MART1 T cells
gp100 T cells
100um
100um
100um
100um
100um
gp100 T cells
100um
0 day
7 days
14 days
MART1 T cells

## Slide 2
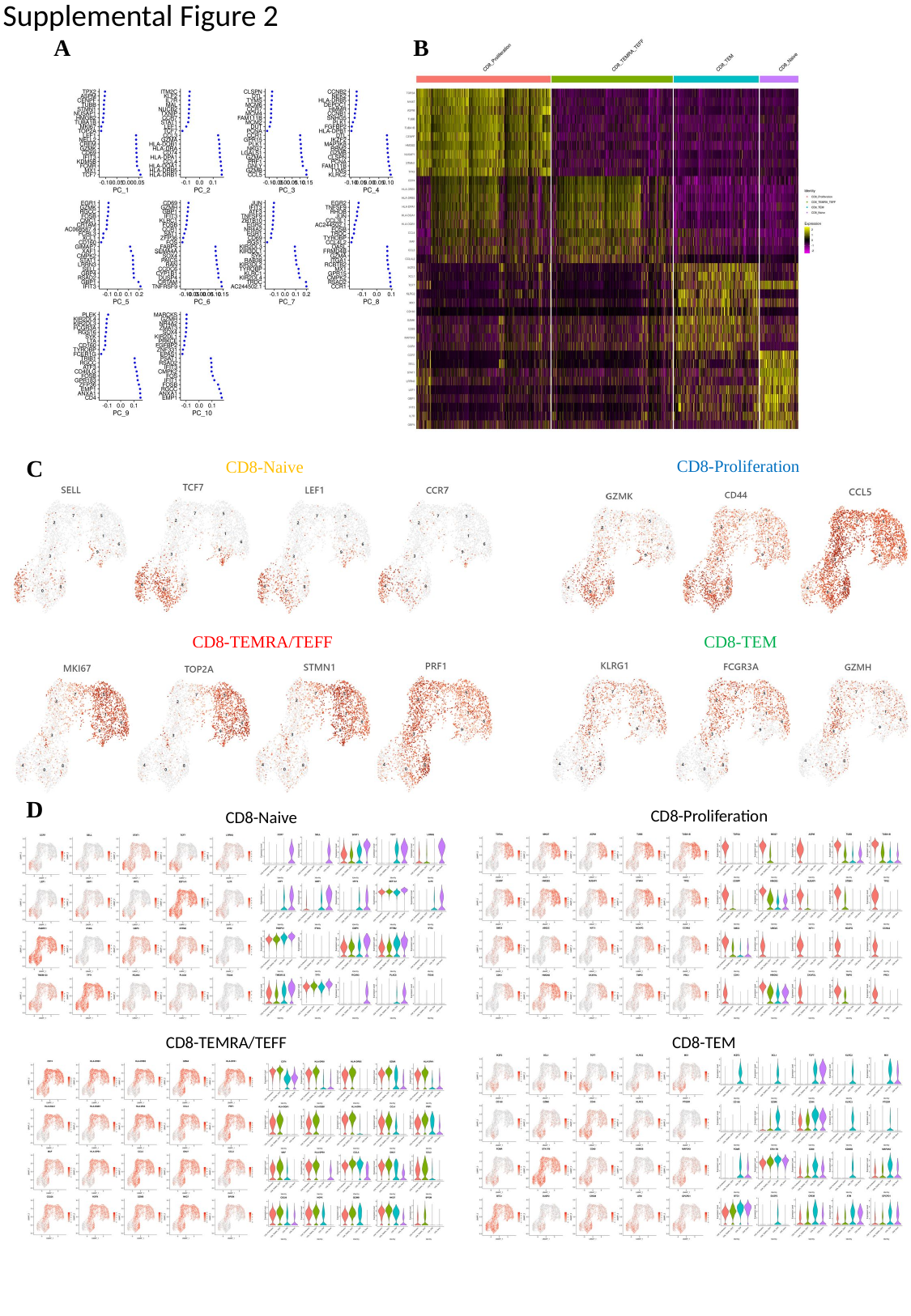

Supplemental Figure 2
A
B
C
CD8-Proliferation
CD8-Naive
CD8-TEM
CD8-TEMRA/TEFF
D
CD8-Proliferation
CD8-Naive
CD8-TEMRA/TEFF
CD8-TEM

## Slide 3
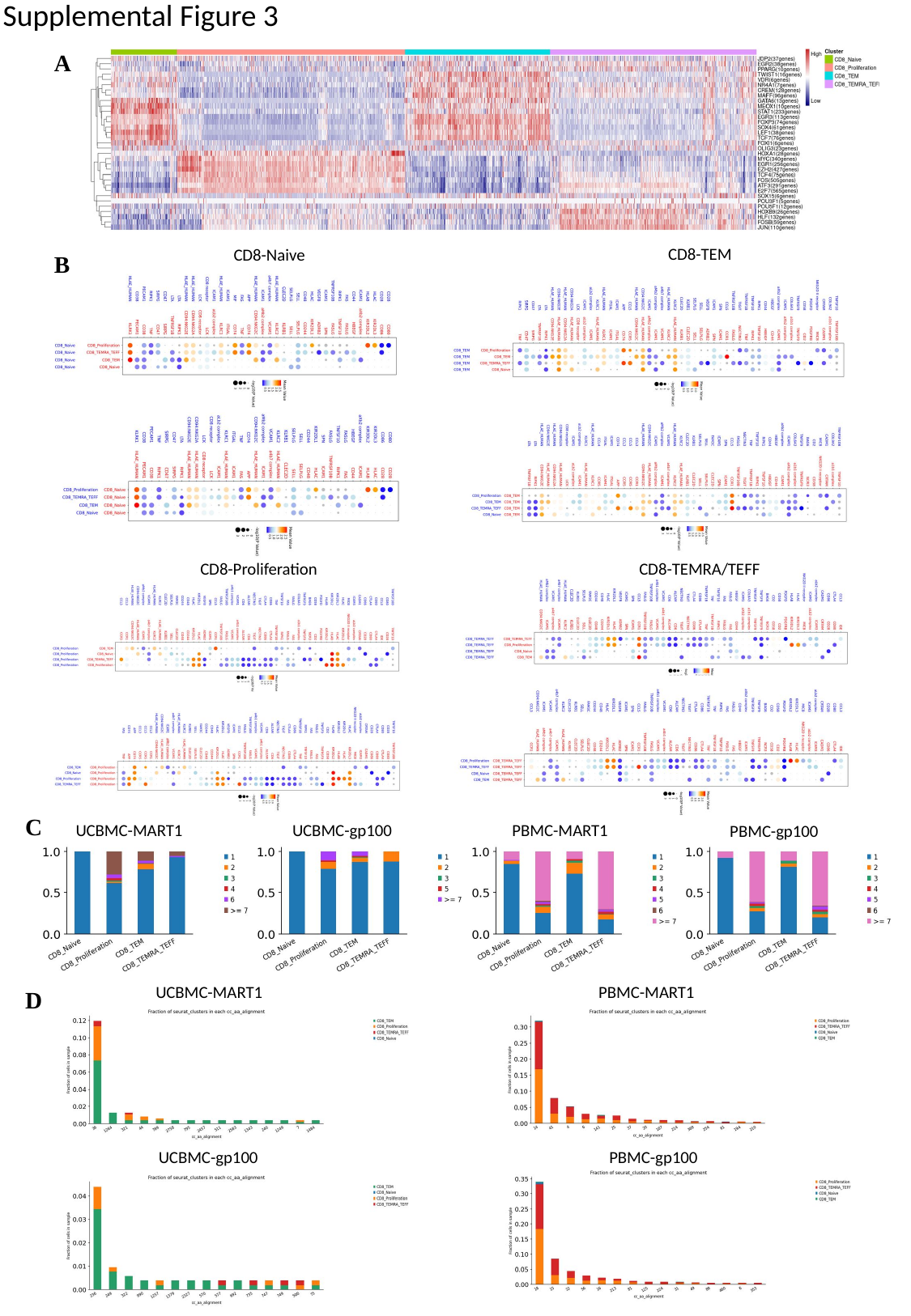

Supplemental Figure 3
A
CD8-TEM
CD8-Naive
B
CD8-TEMRA/TEFF
CD8-Proliferation
C
UCBMC-MART1
PBMC-MART1
UCBMC-gp100
PBMC-gp100
UCBMC-MART1
PBMC-MART1
D
PBMC-gp100
UCBMC-gp100

## Slide 4
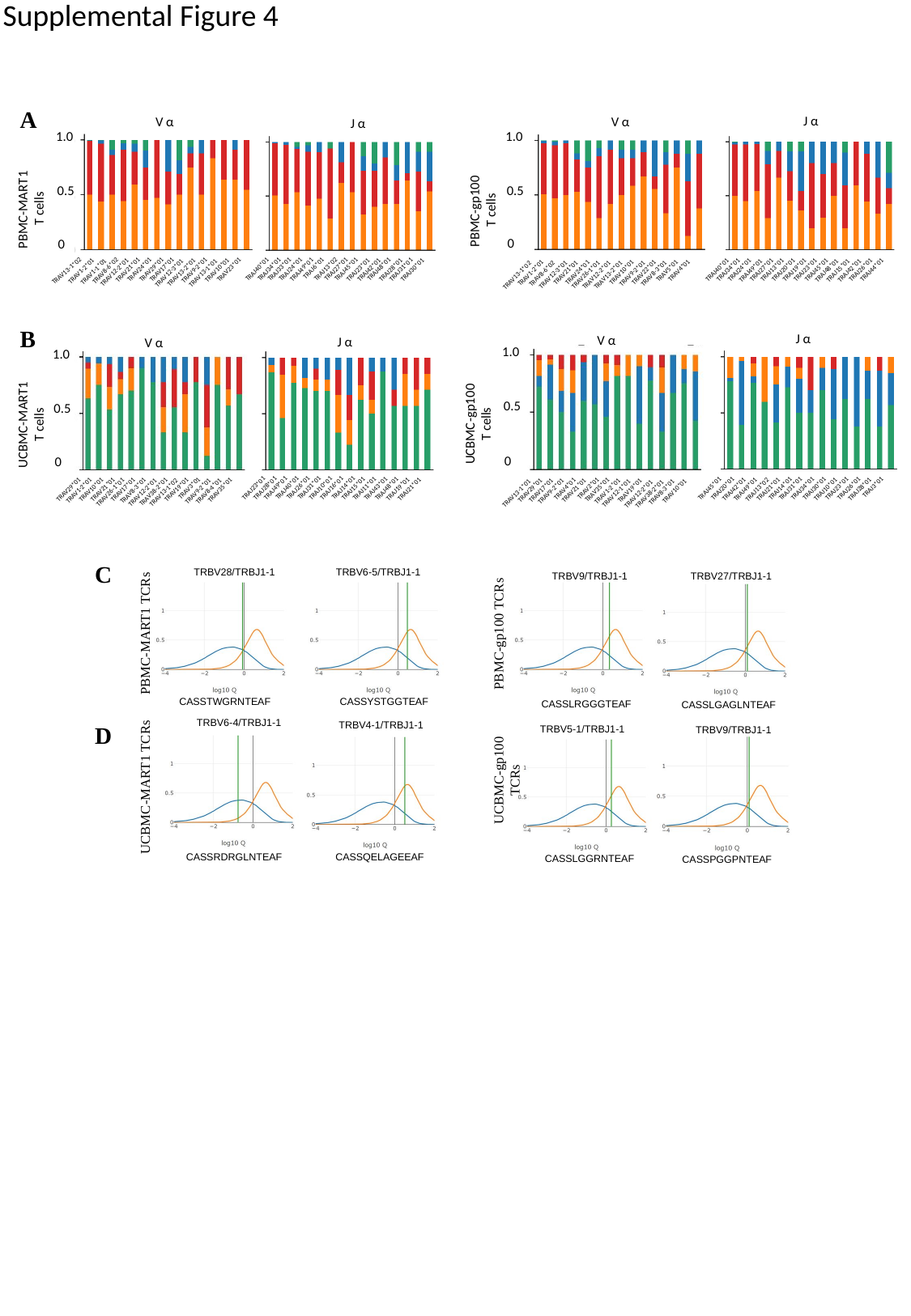

Supplemental Figure 4
A
J α
TRAJ27*01
TRAJ20*01
TRAJ19*01
TRAJ23*01
TRAJ45*01
TRAJ44*01
TRAJ34*01
TRAJ24*01
TRAJ40*01
TRAJ13*01
TRAJ26*01
TRAJ42*01
TRAJ15*01
TRAJ48*01
TRAJ49*01
V α
1.0
0.5
0
TRAV5*01
TRAV22*01
TRAV10*01
TRAV21*01
TRAV24*01
TRAV1-2*01
TRAV8-3*01
TRAV9-2*01
TRAV8-6*02
TRAV26-1*01
TRAV12-3*01
TRAV13-2*01
TRAV12-2*01
TRAV13-1*02
TRAV4*01
V α
1.0
0.5
0
TRAV17*01
TRAV24*01
TRAV29*01
TRAV23*01
TRAV21*01
TRAV9-2*01
TRAV8-6*02
TRAV1-2*01
TRAV1-1*01
TRAV13-1*02
TRAV12-2*01
TRAV13-2*01
TRAV13-1*01
TRAV12-3*01
TRAV10*01
J α
TRAJ6*01
TRAJ27*01
TRAJ48*01
TRAJ13*02
TRAJ45*01
TRAJ24*01
TRAJ33*01
TRAJ34*01
TRAJ49*01
TRAJ31*01
TRAJ42*01
TRAJ23*01
TRAJ30*01
TRAJ28*01
TRAJ40*01
PBMC-MART1
T cells
PBMC-gp100
T cells
B
J α
TRAJ3*01
TRAJ30*01
TRAJ23*01
TRAJ26*01
TRAJ20*01
TRAJ34*01
TRAJ14*01
TRAJ45*01
TRAJ31*01
TRAJ28*01
TRAJ21*01
TRAJ42*01
TRAJ10*01
TRAJ49*01
TRAJ13*02
V α
1.0
0.5
0
TRAV2*01
TRAV4*01
TRAV35*01
TRAV29*01
TRAV21*01
TRAV17*01
TRAV19*01
TRAV10*01
TRAV1-2*01
TRAV9-2*01
TRAV8-3*01
TRAV12-1*01
TRAV13-1*01
TRAV12-2*01
TRAV38-2*01
J α
TRAJ40*01
TRAJ26*01
TRAJ31*01
TRAJ14*01
TRAJ10*01
TRAJ16*01
TRAJ49*01
TRAJ28*01
TRAJ15*01
TRAJ11*01
TRAJ43*01
TRAJ48*01
TRAJ19*01
TRAJ23*01
TRAJ21*01
V α
1.0
0.5
0
TRAV3*01
TRAV21*01
TRAV19*01
TRAV10*01
TRAV29*01
TRAV17*01
TRAV35*01
TRAV1-2*01
TRAV8-3*01
TRAV8-4*01
TRAV9-2*01
TRAV38-2*01
TRAV26-1*01
TRAV13-1*02
TRAV12-2*01
UCBMC-gp100
T cells
UCBMC-MART1
T cells
C
TRBV28/TRBJ1-1
TRBV6-5/TRBJ1-1
CASSTWGRNTEAF
CASSYSTGGTEAF
TRBV9/TRBJ1-1
TRBV27/TRBJ1-1
CASSLRGGGTEAF
CASSLGAGLNTEAF
PBMC-gp100 TCRs
PBMC-MART1 TCRs
TRBV6-4/TRBJ1-1
TRBV4-1/TRBJ1-1
CASSRDRGLNTEAF
CASSQELAGEEAF
D
TRBV5-1/TRBJ1-1
TRBV9/TRBJ1-1
CASSLGGRNTEAF
CASSPGGPNTEAF
UCBMC-gp100 TCRs
UCBMC-MART1 TCRs
